# Improved tolerance of apple plants to drought stress and nitrogen utilization by modulating the rhizosphere microbiome *via* melatonin and dopamine

**DOI:** 10.3389/fmicb.2022.980327

**Published:** 2022-11-10

**Authors:** Peihua Du, Yang Cao, Baoying Yin, Shasha Zhou, Zhongyong Li, Xueying Zhang, Jizhong Xu, Bowen Liang

**Affiliations:** College of Horticulture, Hebei Agricultural University, Baoding, China

**Keywords:** drought, melatonin, dopamine, nitrogen utilization, microbiome, apple

## Abstract

This study explored the contributions of melatonin and dopamine to the uptake and utilization of nitrogen and the formation of rhizosphere microbial communities in ‘Tianhong 2’/*M*. *hupehensis*, with the goal improving plant resistance to drought stress. Drought stress was formed by artificially controlling soil moisture content. And melatonin or dopamine solutions were applied to the soil at regular intervals for experimental treatment. After 60 days of treatment, plant indices were determined and the structure of the rhizosphere microbial community was evaluated using high-throughput sequencing technology. The findings revealed two ways through which melatonin and dopamine alleviate the inhibition of growth and development caused by drought stress by promoting nitrogen uptake and utilization in plants. First, melatonin and dopamine promote the absorption and utilization of nitrogen under drought stress by directly activating nitrogen transporters and nitrogen metabolism-related enzymes in the plant. Second, they promote the absorption of nitrogen by regulating the abundances of specific microbial populations, thereby accelerating the transformation of the soil nitrogen pool to available nitrogen that can be absorbed directly by plant roots and utilized by plants. These findings provide a new framework for understanding how melatonin and dopamine regulate the uptake and utilization of nitrogen in plants and improve their ability to cope with environmental disturbances.

## Introduction

Drought-induced stress, which has been exacerbated by global climate change, has become a critical constraint on global agricultural yields and a threat to global food security (Zhu, 2016; Berdugo et al., 2020). As sessile organisms, most plants are unable to acquire water from long distances and so develop unique strategies to withstand stress associated with aridity (Gupta et al., 2020). However, the capacity of plants to regulate drought stress has a certain threshold; when this is exceeded plant productivity will drop (Berdugo et al., 2020). Although storage of rainwater, water-saving irrigation, mulching, and other cultivation techniques are used to cope with the water shortage caused by drought stress, it is still difficult to solve problems due to long-term drought. This encourages further study of drought-resistance mechanisms and highlights the urgent need for new and effective methods to improve plant drought resistance.

Maintaining nutrient uptake and usability under abiotic stress is an effective means of improving plant stress resistance (Li et al., 2016). Nitrogen (N) is the element that plants need the most, and the application of N fertilizers has been an important measure for increasing agricultural production for a long time (Forde, 2014; Shi et al., 2015). The element also plays a significant role in plant stress resistance (Wang and Macko, 2011). Moreover, studies have revealed that different forms of inorganic N have different effects in the drought tolerance mechanisms of plants. NO_3_^−^ improves the transduction of the stomatal closing signal and relieves photosystem stress under drought conditions (Yi et al., 2014). Application of appropriate amounts of NH_4_^+^ significantly alleviates drought stress (Huang et al., 2018).

Soil holds the N reserve for plant growth and development, and the rhizosphere is an important region of exchange of materials between plants and their surrounding environment. The soil microbial community and its corresponding functions are essential soil indices influencing plant stress resistance and N uptake and utilization (Wang et al., 2020a; Santos-Medellin et al., 2021). With the rapid development of molecular and microbiome technologies, high-throughput sequencing has partially revealed the ways in which the soil microbial community is affected by drought stress and how the community interacts with plants (Prosser, 2020; Wang et al., 2020b). Recent studies have shown the importance of improving plant tolerance to drought in arid ecosystems by utilizing symbiotic relationships with rhizosphere microbial communities, which promote plant nutrient uptake and utilization (Xu et al., 2018). Beneficial microbes of the rhizosphere can improve plant growth and help crop plants cope with increasing drought stress. Although the complex feedback relationships between plant and microbial responses to drought have been studied in depth, most studies have considered non-crop plants (de Vries et al., 2020). The role played in drought regulation by the rhizosphere microbial community of apple and its relationships to N metabolism in the plant continues to be only partially understood.

Dopamine and melatonin are important active antioxidant substances in plants. They participate in the mediation of many physiological and biochemical processes in plant growth and development, and have become high-interest substances in current research on plant resistance to stress (Kanwar et al., 2018; Arnao and Hernández-Ruiz, 2019; Liu et al., 2020). They both significantly promote N uptake and metabolism in plants under drought stress (Liang et al., 2018a,b). For example, they promote the uptake and utilization of NO_3_^−^ and NH_4_^+^ in plants under low N stress (Du et al., 2022a,b). In addition, they improve the rhizosphere microsphere environment, including soil physical and chemical properties, enzyme activity, and rhizosphere microbial community structure, and also promote nutrient absorption and utilization, thus alleviating the inhibition of plant growth and development under repeated stress (Li et al., 2018; Gao et al., 2021). Furthermore, dopamine can promote mycorrhizal colonization in the roots of plants and thus promote the absorption and utilization of nutrients (Gao et al., 2020). However, the comprehensive regulation mechanisms of melatonin and dopamine affecting the apple soil rhizosphere microsphere environment and apple N uptake and utilization under drought stress have not been revealed.

In the present study, we applied a long-term moderate drought treatment to potted apple plants for 60 days to explore the regulatory effects of melatonin and dopamine on N absorption and utilization as well as the rhizosphere microdomain environment of the plants. We hypothesized that these compounds would directly regulate N absorption and utilization and also promote it by regulating the structure of the rhizosphere microbial community. These two effects could effectively alleviate the inhibition of drought stress on plant growth and development.

## Materials and methods

### Plant materials and growing conditions

In brief, field experiments were conducted at the Agricultural University of Hebei, Baoding (34°20΄ N, 108°24΄ E), Hebei, China, mainly with a temperate continental monsoon climate. In mid-March 2020, buds of ‘Tianhong 2’ were grafted onto 1-year-old rootstock of *Malus hupehensis* and grown in white plastic pots (26.5 × 23 cm) filled with soil from the local farming area. The plants were located in a greenhouse under natural light and temperature conditions. To eliminate position effects, we rotated the containers weekly. Weeds and pests were carefully controlled during the experiment.

### Experimental designations

All plants were pre-cultured under sufficient water for 3 months. Then the plants were divided into the following six experimental groups: soil moisture content 75–85% (CK); moderate drought, soil moisture content 45–55% (DT); soil moisture content 75–85% plus 100 μmol/l melatonin (MCK); soil moisture content 45–55% plus 100 μmol/l melatonin (MDT); soil moisture content 75–85% plus 100 μmol/l dopamine (DCK); soil moisture content 45–55% plus 100 μmol/l dopamine (DDT). Transpiration water losses were evaluated gravimetrically by weighing all pots and calculating the changes in weight that occurred between watering events. Afterward, the amount of water lost was added back to each pot every other day at 18: 00 h. For half of the plants in either the well-watered or moderate drought treatments, exogenous melatonin or dopamine was applied with a 100 μmol/L solution replacing the same amount of water added back to the soil every 10 days. The experimental plot (water and melatonin/dopamine treatments) was completely randomly distributed. Each group was treated with 50 replicates. Ten plants were randomly selected and treated with 1.5 g CO (^15^NH_2_)_2_, and the rest were treated with 1.5 g normal urea. The test treatment was carried out in the greenhouse from July 15, 2020 and lasted for 60 days.

### Plant growth and physiological indicators

At the end of the experiment, plant length (PL) and stem diameter (TD) were measured using a straight ruler and Vernier calipers according to the determination standards of a previous study (Liang et al., 2018b). Then the whole plants were divided into roots, stems, and leaves, which were cleaned with tap water and deionized water, followed by heating at temperatures up to 105°C for 15 min and continuous drying at 65°C to constant weight. Total dry weight (TDW) was calculated by summing the root dry weight, stem dry weight, and leaf dry weight, and the relative growth rate (RGR) was measured according to the formula described in a previous study (Liang et al., 2018b). The total chlorophyll content was determined using the 80% acetone colorimetric method (Li et al., 2018).

Superoxide dismutase (SOD) was determined using the photochemical nitroblue tetrazolium method. Peroxidase (POD) was determined using the guaiacol method. Catalase was determined using the ultraviolet absorption method (Balfagon et al., 2021). The enzyme activities were determined by referring to the three aforementioned enzyme activity determination methods (Ahmad et al., 2020). According to the instructions provided with the kit (Comin Biotechnology Co., Suzhou, China), 0.1 g fresh leaves were taken and 1 ml extract was added for ice grinding extraction. After centrifugation, spectrophotometry was used to determine ascorbate peroxidase (APX).

The content of malondialdehyde (MDA) was determined using the thiobarbituric acid method: 1.6 ml 10% trichloroacetic acid (TCA) was added to 0.2 g leaves. The soluble sugar was measured using the anthrone method using 1 g leaves. Free proline was extracted with 0.2 g leaves and 3% sulfosalicylic acid. The three aforementioned determination methods followed a previous study (Sharma et al., 2020). The production rate of superoxide anion in plant leaves was determined using a previously described method (Tian et al., 2003).

### Measurements of melatonin and dopamine contents

Leaf samples for extraction of melatonin and dopamine were taken after 60 days of treatment. The melatonin content was measured according to the method described in our previous study (Liang et al., 2018b). And the method of determining dopamine content was taken from Gao et al. (2020).

### N metabolism and transport in the plants

The δ^15^N content and determination method, the absorption and utilization efficiency, and related calculation formulae were referenced from a previous study (Liang et al., 2017). The nitrate reductase (NR), nitrite reductase (NiR), glutamine synthetase (GS), and ferredoxin-dependent glutamate synthase (Fd-GOGAT) activities in leaves were determined using the relevant kits (Comin Biotechnology Co., Suzhou, China).

According to the instructions, the total RNA from the plant leaf samples was extracted using the M5 Plant RNeasy Complex Mini Kit (Mei5 Biotechnology, Co., Ltd., Beijing, China). Then the RNA was reverse-transcribed into cDNA using the UEIris RT-PCR System for the First-strand cDNA Synthesis System (US Everbright Inc., Suzhou, China). Finally, using the Roche LightCycler 96 real-time PCR System (Roche, Basel, Switzerland), N metabolism and *AMT* and *NRT* gene expression levels were detected using the primers in Supplementary Table S1. *β-actin* was the internal reference gene in the experiment.

### Soil sampling and measurements

All necessary tools were disinfected before the experiment. Four replicates were set for each treatment, and 10 plants were mixed for each replicate. First, the plant roots were taken out, and the soil around the rhizosphere was removed by shaking. Then the soil of the rhizosphere was scraped with a blade, impurities were removed with a 2 mm sieve, and the soil was divided into three parts. Soil samples for microbial determination were first placed in sterile tubes and immediately fixed in liquid nitrogen. In addition, the remaining corresponding soil samples were placed in coolers and returned to the laboratory in a timely manner. A portion of the soil samples was stored in a 4°C refrigerator and used to determine the microbial biomass C (MBC) and microbial biomass N (MBN); the other portion was dried naturally to determine the physical and chemical properties of the soil. Soil physical and chemical properties, including available nitrogen (AN), available phosphorus (AP), available potassium (AK), pH, and soil organic matter (SOM), were determined using standardized soil testing procedures (Wang et al., 2020b). The MBC and MBN in the soil were determined *via* chloroform fumigation (Valverde et al., 2016). Soil urease (UR) was determined using a previously described method (Ge et al., 2010).

### Microbial community analysis

The HiPure Soil DNA Extraction Kit (Magen, Guangzhou, China) was used according to the instructions to extract microbial DNA from a 0.5 g soil sample. After testing the concentration and purity, the soil microbial DNA was placed in a − 80°C refrigerator until testing. Specific primers with barcodes were used to amplify the ITS2 region of ITS and the V3 + V4 region of 16S rDNA, respectively. The ITS target and 16S rDNA region of the ribosomal RNA gene were amplified by PCR and the primer sequences were as follows: ITS3_KYO2: GATGAAGAACGYAGYRAA; ITS4: TCCTCCGCTTATTG ATATGC; 341F: CCTACGGGNGGCWGCAG; 806R: GGAC TACHVGGGTATCTAAT. Purification was performed using the AxyPrep DNA Gel Extraction Kit (Axygen Biosciences, Union City, CA, United States) and was followed by quantification using the ABI StepOnePlus real-time PCR system (Life Technologies, Foster City, USA). Purified amplicons were sequenced on the Illumina platform according to standard operations (PE250), and the original read data were uploaded to the NCBI Sequence Read Archive (SRA) database (Accession Number: SRP388303). The raw data of the Illumina platform were filtered using FASTP (version 0.18.0; Guo et al., 2017). We removed reads containing more than 10% of unknown nucleotide (N) reads, reads containing less than 50% of quality bases, and values exceeding 20, resulting in clean reads. The clean reads were combined into tags using FLASH (version 1.2.11) at a minimum overlap of 10 bp and a maximum mismatch rate of 2%. Then, low-quality tags were filtered to obtain high-quality clean tags according to the filtering conditions of QIIME software (Bokulich et al., 2013). Finally, effective tags were obtained after the chimera was filtered. The effective tags were clustered into operational taxonomic units (OTUs) using USEARCH software, and the OTU abundance and other analysis results were attained.

### Data visualization and statistical analysis

The RDP Classifier (version 2.2) was used to obtain naive Bayesian model results, and species classification annotation was performed for OTU representative sequences of fungi or bacteria based on the SILVA database (version 132), UNITE database (version 8.0), or ITS2 database. The abundance of each species classification was computed using Krona (version 2.6), and the pattern of species abundance was illustrated using R software. QIIME (version 1.9.1) was used to calculate the alpha diversity index for each microbial treatment community, and the Tukey test was used to compare the diversity index across different treatments (*p* < 0.05). The Bray–Curtis distance matrix was calculated using R based on the OTUs and a species abundance table, and non-metric multidimensional scaling (NMDS) multivariate statistical analysis was performed. The Omicsmart dynamic real-time interactive online data analysis platform^1^ was used to generate heatmaps and correlation network results.

All statistical analysis results for the plants were obtained using IBM SPSS Statistics 20 software (IBM Corp, Armonk, NY, United States). Data visualization was implemented using SigmaPlot 10.0 (Systat Software, Inc., San Jose, CA, United States). All treatments were compared and analyzed using one-way analysis of variance, and significant differences between treatments were determined using Tukey’s multiple-range test (*p* < 0.05).

## Results

### Plant growth and development

**Figure 1:**
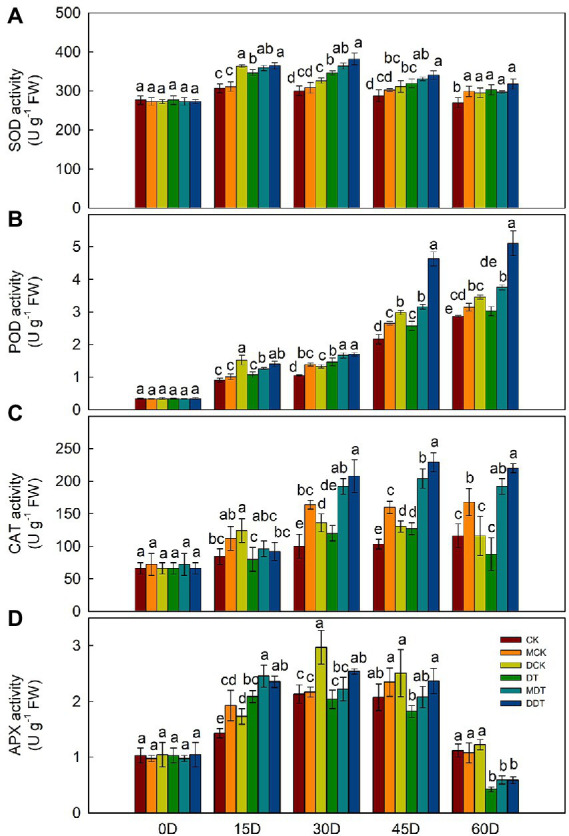
Dynamic changes of antioxidant enzyme activity [**(A)** SOD; **(B)** POD; **(C)** CAT, and **(D)** APX] in leaves under different treatment. Data are means ± SD (*n* = 3). For each panel, bars not labeled with same letter indicate significant differences at *p* < 0.05, based on Tukey’s multiple range tests.

After 60 days of drought treatment, the growth and development of DT were significantly inhibited, but melatonin and dopamine were able to mitigate the stress-inhibiting effect on the plants (Supplementary Figures S1, S2). The MDA content and the production rate of superoxide anion were significantly higher in DT leaves than in CK. Melatonin and dopamine significantly reduced the reactive oxygen and increased the level of free proline and soluble sugar in leaves under drought stress compared to DT (Supplementary Figure S3). In addition, we measured the activities of four antioxidant enzymes (SOD, CAT, POD, and APX) every 15 days. The activities of the enzymes were higher in DT plant leaves than in CK (Figure 1). The activities of SOD, CAT, and APX initially increased and then decreased as the treatment time against drought increased. Melatonin and dopamine further enhanced the activities of the plant’s four antioxidant enzymes compared to DT (Figure 1).

In addition, we measured the levels of melatonin and dopamine in plant leaves after 60 days of treatment. The contents of melatonin and dopamine in DT were increased by 76.36 and 12.24%, respectively, compared to CK. The addition of melatonin and dopamine can result in increased levels of endogenous melatonin and dopamine, particularly in the case of drought. Their levels in MDT and DDT increased by 144.77 and 55.75%, respectively, compared to CK (Supplementary Figure S4).

### Uptake, transport, and metabolism of N in plants

**Figure 2:**
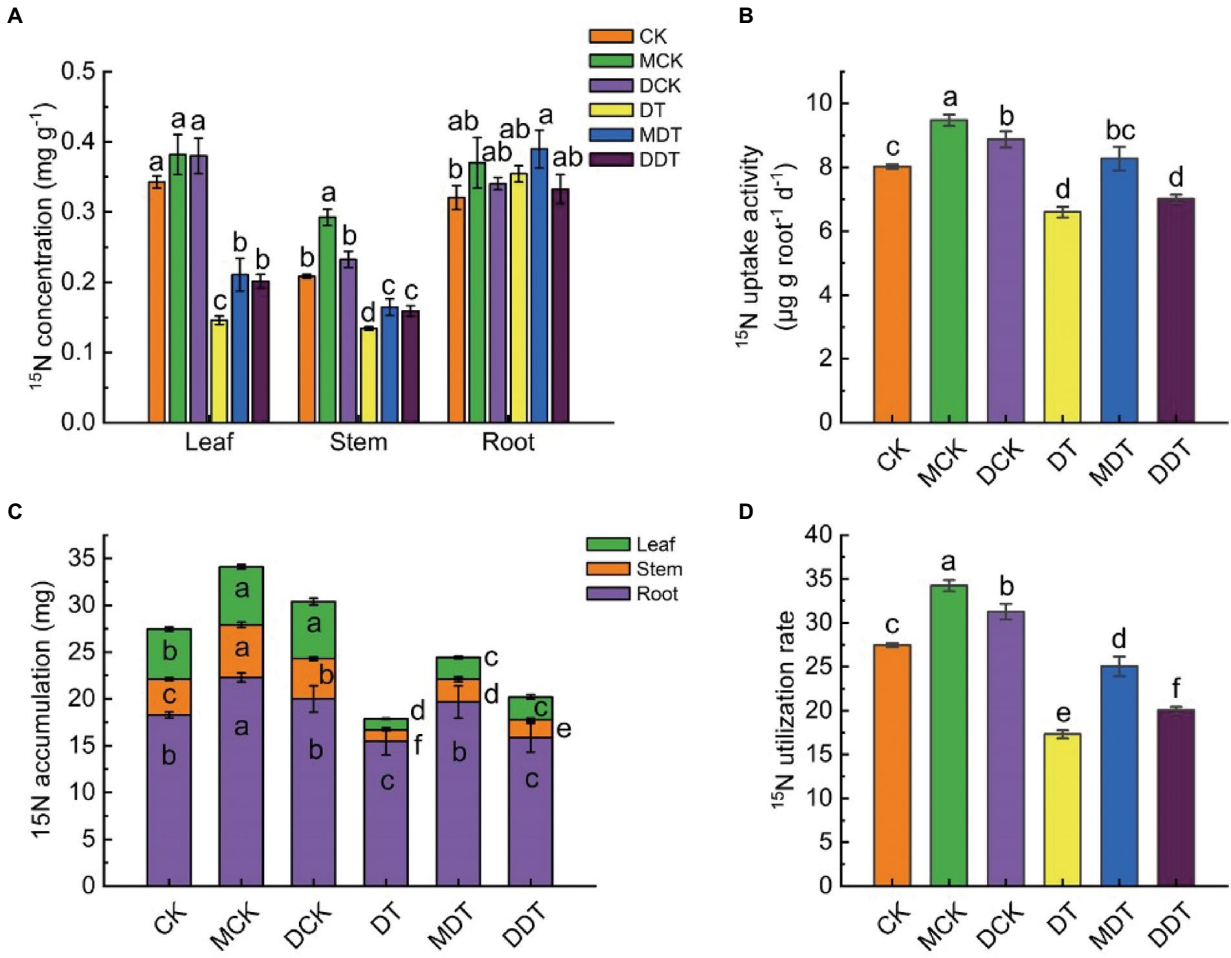
The ^15^N isotope labeling results. ^15^N concentrations **(A)** and accumulation **(C)** in different parts of plant, ^15^N uptake activity **(B)** and utilization rate **(D)**. Data are means ± SD (*n* = 5). For each panel, bars not labeled with same letter indicate significant differences at *p* < 0.05, based on Tukey’s multiple range tests.

The ^15^N isotopic tracer results indicated that the concentration and accumulation of ^15^N in DT leaves and stems declined significantly compared to CK under long-term drought stress (Figures 2A,C). However, there were no significant differences in the concentration of ^15^N between DT roots and CK roots. Melatonin and dopamine significantly increased the concentration of ^15^N in leaves and stems by 44.72 and 38.20% and 22.65 and 18.48%, respectively, compared to DT (Figure 2A). Melatonin and dopamine mitigated the decrease in ^15^N accumulation in leaves, stems, and roots, producing values higher by 88.42 and 98.42%, 106.77 and 64.73%, and 27.08 and 2.26%, respectively, relative to DT (Figure 2C). Drought stress also resulted in significant decreases in the ^15^N absorption capacity and utilization rate of DT of 17.12 and 36.56%, respectively, compared to CK. The ^15^N utilization rate and uptake activity in MDT and DDT increased by 44.36 and 13.22% and 24.91 and 8.23%, respectively, compared to DT (Figures 2B,D).

**Figure 3:**
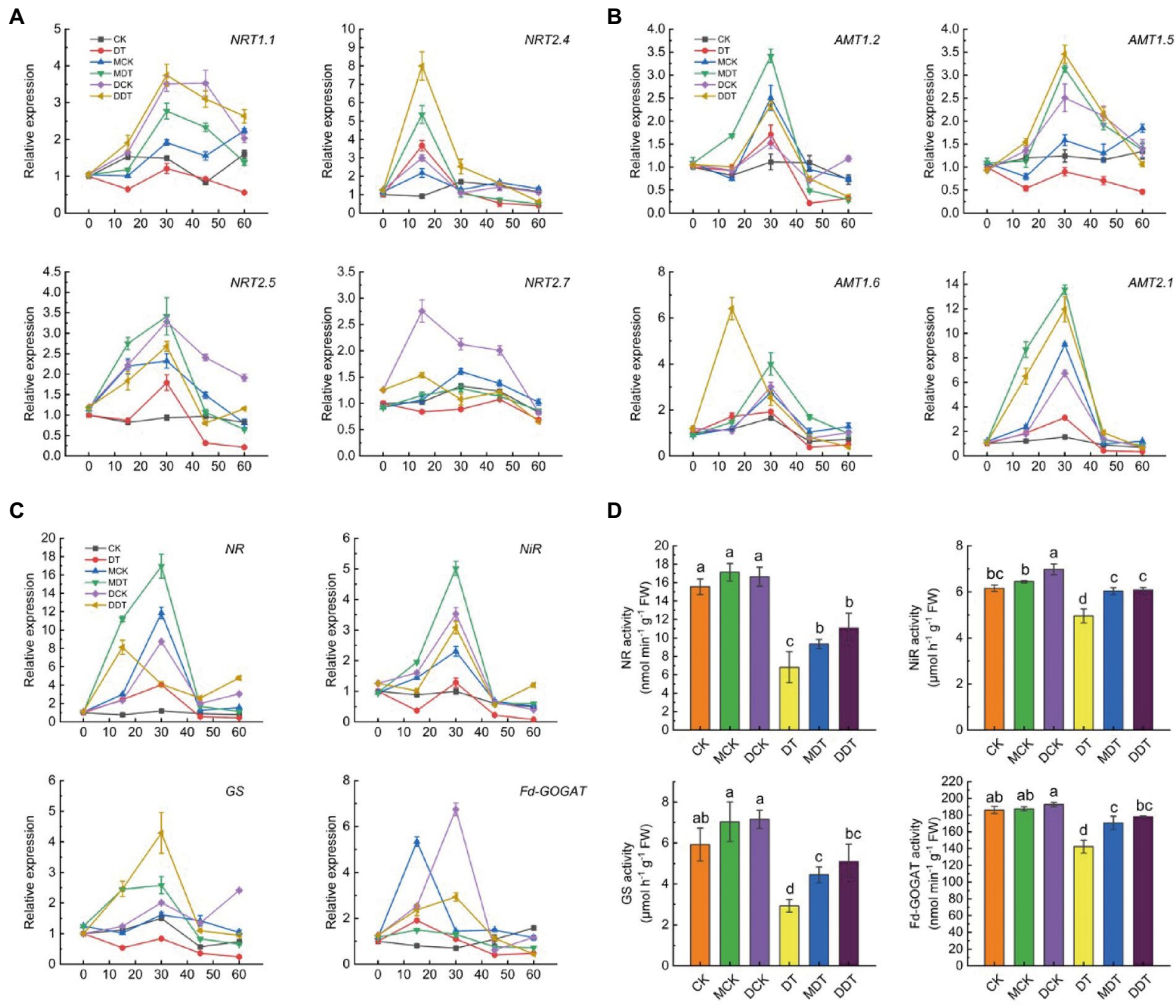
Dynamic changes of N transport [**(A)** *NRTs*: *NRT1.1*, *NRT2.4*, *NRT2.5* and *NRT2.7*; **(B)** *AMTs*: *AMT1.2*, *AMT1.5*, *AMT1.6* and *AMT2.1*] and metabolism (**C**: *NR*, *NiR*, *GS*, and *Fd-GOGAT*) associated genes and final N metabolic enzyme activity **(D)** in leaves under different treatment. Data are means ± SD (*N* = 3). For each panel, bars not labeled with same letter indicate significant differences at *p* < 0.05, based on Tukey’s multiple range tests.

In addition, every 15 days, we determined the relative expressions of *NRT* and *AMT* family fractional genes associated with N uptake and transport in plant leaves subjected to the different treatments (Figure 3). After 30 days of drought treatment, the relative expressions of *NRT1*.*1*, *NRT2*.*4*, *NRT2*.*5*, *NRT2*.*7*, *AMT1*.*2*, *AMT1*.*5*, *AMT1*.*6*, and *AMT2*.*1* in DT leaves began to decrease. Compared to CK 0 days, these genes decreased by 1.79, 2.44, 4.67, 1.65, 3.09, 2.15, 2.02, and 2.81 folds, respectively, after 60 days of treatment (Figures 3A,B). On the contrary, melatonin and dopamine significantly upregulated the expression of *NRTs* and *AMTs* to varying degrees, showing a trend of first increasing and then decreasing them. Among these genes, *NRT1*.*1*, *NRT2*.*5*, *AMT1*.*2*, *AMT1*.*6*, and *AMT2*.*1* reached their peak after 30 days of treatment in MDT and DDT and were significantly upregulated by 3.42 and 2.34, 4.00 and 2.52, and 13.56 and 11.96 folds, respectively, compared to CK 0 days. Moreover, *NRT2*.*4* genes peaked after 15 days of treatment in MDT and DDT and were upregulated by 5.36 and 8.00 folds, respectively (Figures 3A,B).

Meanwhile, the *NR*, *NiR*, *GS*, and *Fd-GOGAT* genes were downregulated in the leaves of DT plants after 60 days of drought stress. Compared to CK on day 0, the *NR*, *NiR*, *GS*, and *Fd-GOGAT* genes were downregulated by 2.15, 12.75, 4.09, and 2.67 folds after 60 days of treatment, respectively (Figure 3C). The expressions of *NR*, *NiR*, *GS*, and *Fd-GOGAT* in MDT and DDT plants first increased and then decreased. The *NiR* and *GS* genes reached their peak at 30 days after treatment and were upregulated by 5.02 and 3.08 and 2.58 and 4.29 folds, respectively (Figure 3C). We also measured N metabolism-related enzyme activities after 60 days. The enzyme activities of NR, NiR, GS, and FD-GOGAT in leaves of DT were significantly decreased by 56.25, 19.52, 50.60, and 23.56%, respectively, compared to CK (Figure 3D). Melatonin or dopamine significantly alleviated the inhibitory effect of drought on N metabolic enzymes. The activities of NR, NiR, GS, and Fd-GAGOT in MDT and DDT plants were increased by 37.14 and 62.38%, 21.81 and 22.62%, 52.00 and 73.90%, and 19.91 and 25.19%, respectively, compared to DT (Figure 3D).

### Soil physicochemical properties and microbial community diversity

After 60 days of drought treatment, the contents of AN, AP, AK, MBC, and MBN in DT rhizosphere soil were significantly decreased by 22.61, 17.38, 25.32, 26.89, and 43.18%, respectively, compared to CK (Table 1). Melatonin and dopamine significantly alleviated the decline of rhizosphere soil nutrient content, which decreased by only 8.65 and 16.36%, 8.75 and 9.39%, 13.44 and 15.50%, 0.01 and 12.90%, and 28.47 and 27.37% compared to CK (Table 1). The urease activity increased significantly by 13.75 and 5.50% in MDT and DDT, respectively, compared to DT (Table 1).

**Table 1:** Effects of melatonin and dopamine on available nitrogen (AN), available phosphorus (AP), available potassium (AK), soil organic matter (SOM), pH, soil microbial biomass C (MBC) and N (MBN), and urease activity under long-term drought stress.

Treatment	AN (mg/kg)	AP (mg/kg)	AK (mg/kg)	SOM (g/kg)	pH	MBC (mg/kg)	MBN (mg/kg)	Urease activity (mg/g/d)
CK	90.45 ± 2.77 a	294.13 ± 13.89 a	580.50 ± 21.00 a	56.15 ± 0.90 b	8.23 ± 0.16 a	274.97 ± 8.79 a	23.85 ± 2.49 a	1.67 ± 0.01 c
DT	70.00 ± 1.37 d	243.00 ± 6.83 c	433.50 ± 26.10 c	63.84 ± 2.47 a	8.29 ± 0.14 a	201.02 ± 9.42 b	13.55 ± 0.66 c	1.73 ± 0.04 c
MDT	82.63 ± 2.06 b	268.38 ± 7.60 b	502.50 ± 19.82 b	66.30 ± 3.92 a	8.22 ± 0.11 a	274.93 ± 41.11 a	17.06 ± 1.11 b	1.97 ± 0.07 a
DDT	75.65 ± 2.74 c	266.50 ± 10.65 b	490.50 ± 36.78 b	68.26 ± 0.90 a	8.30 ± 0.09 a	239.51 ± 1.71 ab	17.32 ± 0.17 b	1.83 ± 0.03 b

Data are means ± SD (*n* = 4). Different letters indicate significant differences at *p* < 0.05 (Tukey’s multiple-range test).

**Figure 4:**
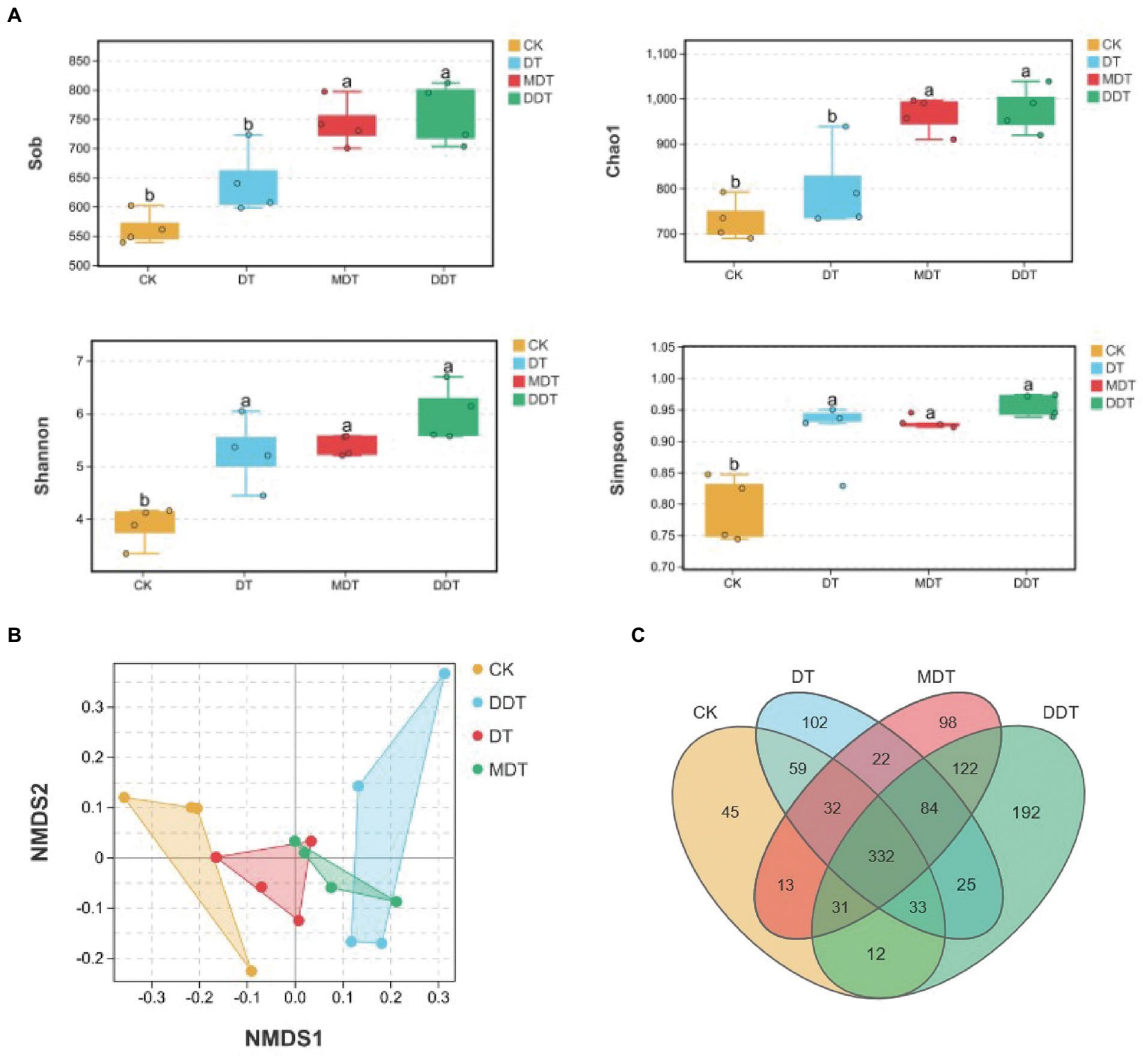
Rhizosphere soil fungal community diversity [**(A)** α-diversity: Sob, Chao1, Shannon, Simpson; **(B)** β-diversity: NMDS)] and OTU number **(C)** under different treatments.

**Figure 5:**
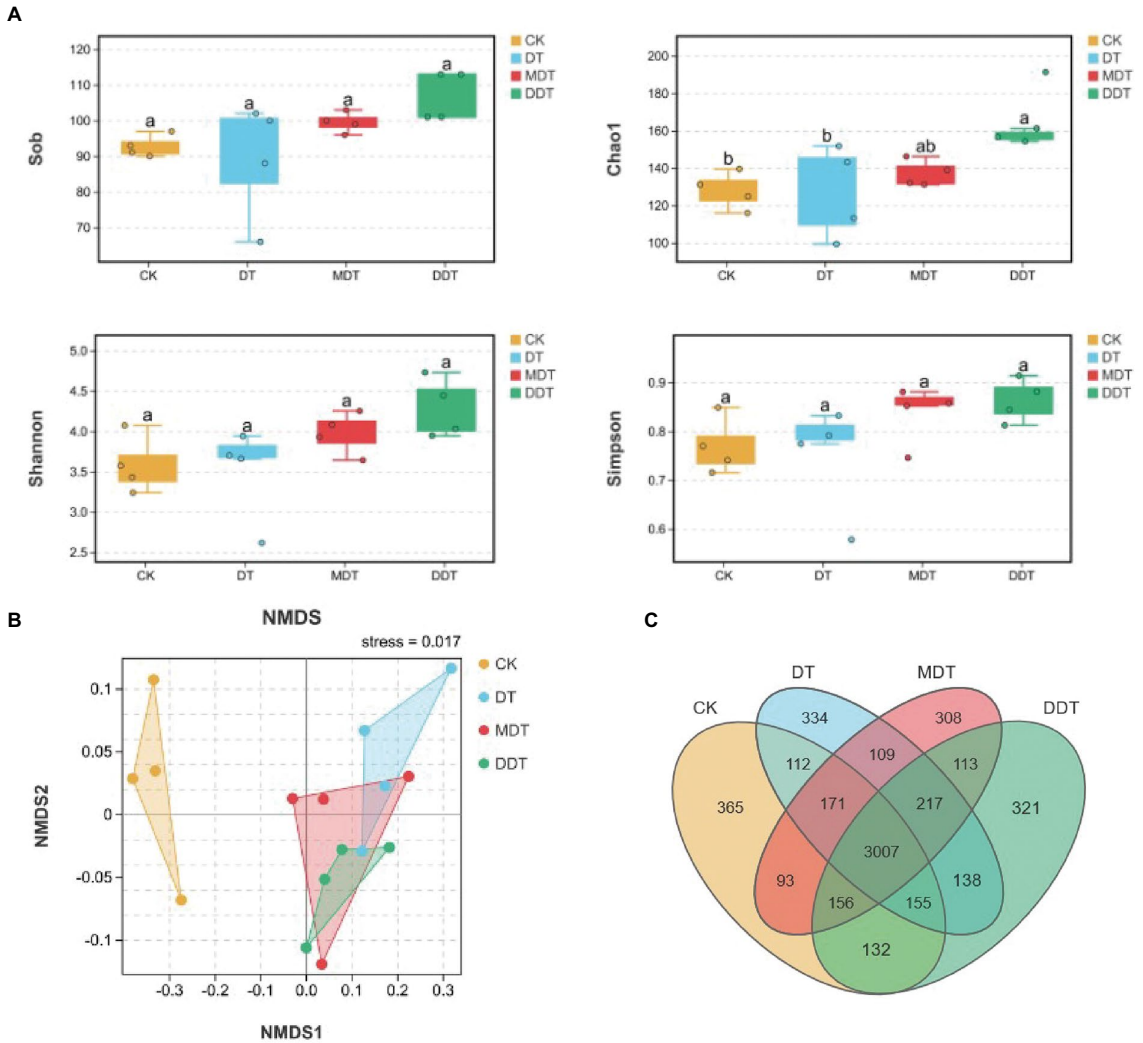
Rhizosphere soil bacterial community diversity **(A)** α-diversity: Sob, Chao1, Shannon, Simpson; **(B)** β-diversity: NMDS) and OUT number **(C)** under different treatments.

Quality screening of 16S rDNA and ITS sequences from 24 soil samples resulted in 1,202 fungal OTUs and 5,731 bacterial OTUs, respectively (Figure 4C). Moreover, long-term moderate drought significantly increased the OTU number of fungi compared to CK. Moreover, the numbers of fungi OTUs in MDT and DDT were significantly higher than the number in DT (Figure 4C). There were no significant differences in the OTU numbers of bacteria (Figure 5C). Compared to CK, the Simpson and Shannon indices of the fungal community were significantly increased in DT, MDT, and DDT. The Sob and Chao1 were significantly higher in MDT and DDT than DT (Figure 4A). There were no significant differences in the α diversity of the bacterial communities among the four treatments (Figure 5A). NMDS analysis based on Bray-Curtis distance was performed to compare similarities or differences in fungal and bacterial community composition between the four treatments at the OTU level. The results showed that the soil fungal and bacterial communities of the four treatments were separated to various degrees (Figures 4B, 5D).

In addition, the comprehensive analysis of the interaction between soil fungal and bacterial communities showed that the Sob, ACE, and Shannon results of 16S/ITS were lower than those of CK after long-term drought treatment and decreased further after melatonin and dopamine were applied (Supplementary Figure S5). The number of negative correlations between fungal and bacterial communities was much higher than the number of positive correlations, indicating that there was strong competition between them (Supplementary Figure S5).

### Microbial community structure

**Figure 6:**
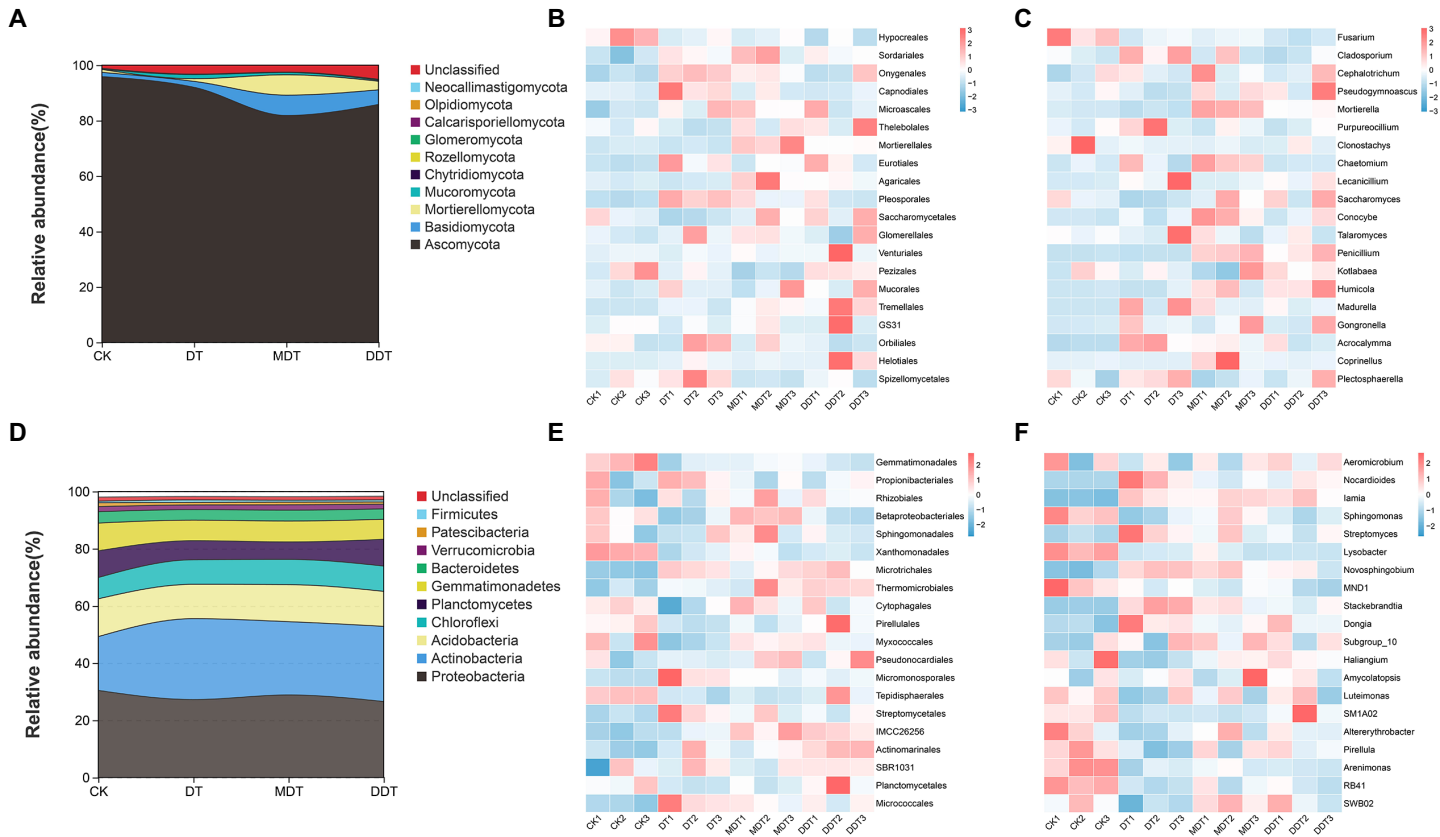
Phyla, order and genus horizontal community structure of rhizosphere soil fungi **(A–C)** and bacteria **(D–F)**.

The relative abundances of phyla, orders, and genera of soil fungi and bacteria differed under the four treatments (Figure 6). At the phylum level of fungi, Ascomycota was dominant (81.86–95.88%). After long-term drought treatment, the relative average abundance of Chytridiomycota was significantly reduced compared to CK. Melatonin and dopamine significantly increased the relative average abundance of Basidiomycota and Mortierellomycota compared to DT (Figure 6A). Proteobacteria (26.56–30.45%) and Actinobacteria (18.81–28.30%) were the dominant groups of soil bacteria at the phylum level. After long-term drought treatment, the relative average abundances of Proteobacteria, Planctomycetes, Gemmatimonadetes, and Patescibacteria in rhizosphere were decreased significantly compared to CK. The average relative abundance of Actinobacteria increased significantly compared to CK after long-term drought treatment. Dopamine significantly increased the relative average abundance of Proteobacteria compared to DT. However, melatonin did not significantly affect the composition of the bacterial microbial community at the phylum level compared to DT (Figure 6D).

Moreover, heatmaps of species abundance revealed differences in fungal and bacterial community composition at the taxonomic levels of order and genus under the four treatments (Figure 6). At the order level of fungi, the average relative abundance of Hypocreales was decreased significantly and the average abundances of Onygenales, Capnodiales, Eurotiales, Pleosporales, and Spizellomycetales were increased after drought treatment compared to CK (Figure 6B). Melatonin reduced the relative average abundances of Capnodiales, Pleosporales, Pezizales, and Spizellomycetales and increased the abundances of Mortierellales and Agaricales compared to DT. Dopamine reduced the relative average abundances of Onygenales, Capnodiales, Pleosporales, and Spizellomycetales and increased the relative average abundances of Saccharomycetales and Tremellales compared to DT (Figure 6B). At the genus level of fungi, the relative average abundance of *Fusarium* was significantly lower in DT than in CK and the relative average abundance of *Cladosporium* was significantly higher (Figure 6C). Melatonin improved the relative average abundances of *Conocybe*, *Mortierella*, and *Penicillium* and reduced the relative average abundances of *Cladosporium*, *Purpureocillium*, and *Gongronella* compared to DT. Dopamine increased the relative average abundances of *Mortierella*, *Penicillium*, and *Humicola* and reduced the relative average abundance of *Acrocalymma* compared to DT (Figure 6C).

At the order level of bacteria, the relative average abundances of Gemmatimonadales, Betaproteobacteriales, Xanthomonadales, Myxococcales, and Tepidisphaerales were significantly decreased and the relative average abundances of Microtrichales, Micromonosporales, Streptomycetales, Actinomarinales, and Micrococcales were significantly increased under drought treatment compared to CK (Figure 6E). Melatonin reduced the relative average abundance of Micromonosporales and increased the abundances of Betaproteobacteriales, Thermomicrobiales, and IMCC26256 compared to DT. Dopamine reduced the relative average abundance of Micromonosporales and increased the relative average abundances of Thermomicrobiales and IMCC26256 compared to DT (Figure 6E). Moreover, at the genus level of bacteria, DT decreased the relative average abundances of *Sphingomonas*, *Lysobacter*, *Novosphingobium*, *MND1*, *Stackebrandtia*, *Haliangium*, *Pirellula*, *Arenimonas*, *RB41*, and *SWB02* and increased the relative average abundances of *Nocardioides*, *Iamia*, *Streptomyces*, *Novosphingobium*, *Stackebrandtia*, and *Dongia* compared to CK. Melatonin reduced the relative average abundances of *Stackebrandtia* and *Nocardioides* and increased the relative average abundance of *Sphingomonas* compared to DT. Dopamine reduced the relative average abundances of *Nocardioides*, *Novosphingobium*, and *Stackebrandtia* compared to DT (Figure 6F).

### Network of interactions among environment, plant, and microorganisms

**Figure 7:**
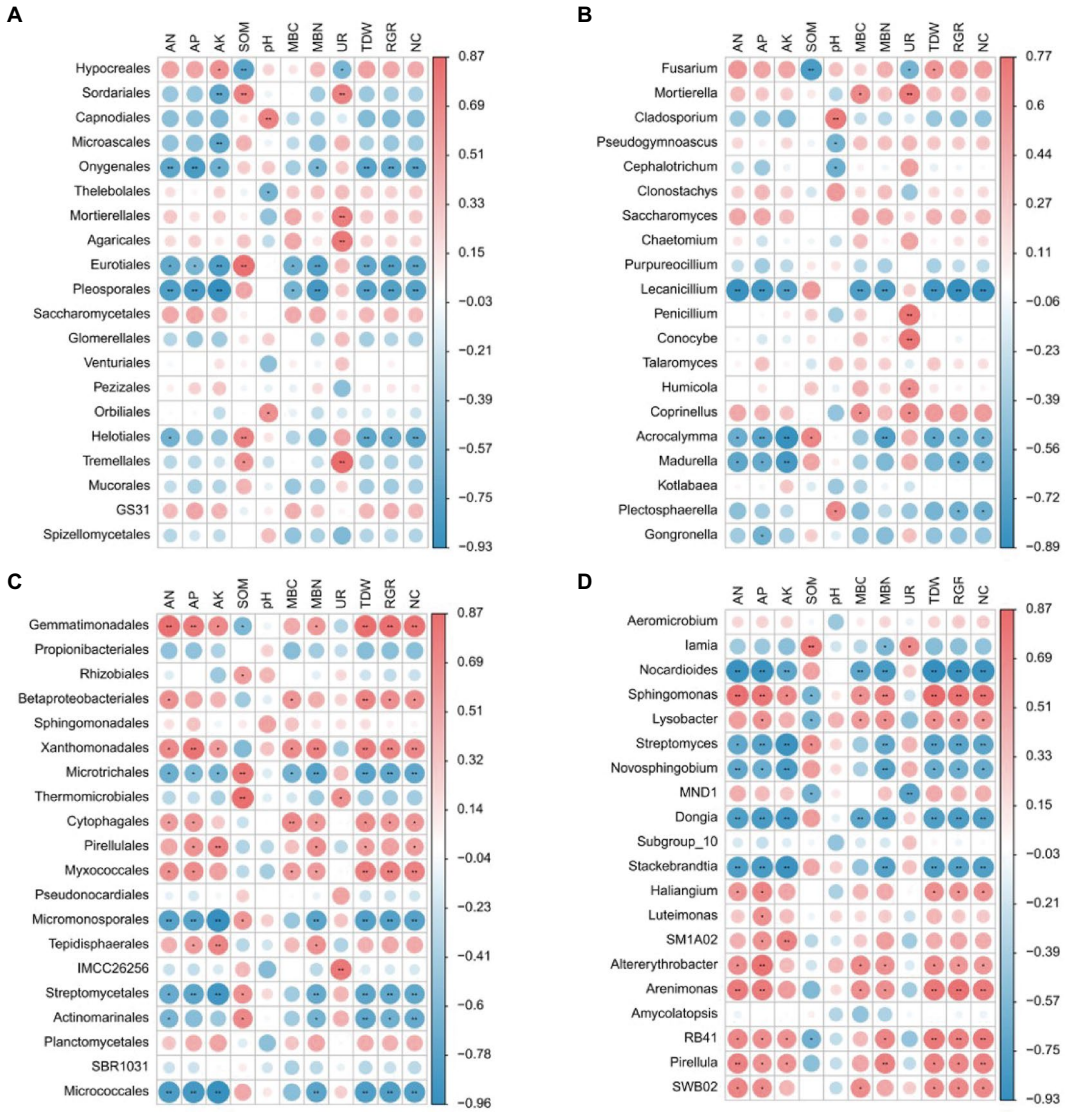
Spearman correlations between the relative abundance of microbial community [**(A)** the dominant fungal order; **(B)** fungal genus; **(C)** bacterial order; **(D)** bacterial genus] and environmental factor (edaphic indicator and plant indicator). The red color indicates positive correlations, while the blue color indicates negative correlations (*p < 0.05, **p < 0.01; Spearman).

To obtain an in-depth understanding of the bacterial community responses to plant growth and development, N uptake, and rhizosphere soil conditions, further correlation analyses were performed among eight soil indices (AN, AP, AK, SOM, PH, MBC, MBN, and UR), three plant indices (TDW, RGR, NC: ^15^N content), and fungal or bacterial orders and genera (Figure 7). At the order level of fungi, Hypocreales correlated positively with AK and Tremellales correlated positively with UR. Onygenales, Eurotiales, and Pleosporales correlated negatively with AN, AP, AK, MBC, MBN, TDW, RGR, and NC (Figure 7A). At the genus level of fungi, *Mortierella*, *Penicillium*, *Humicola*, and *Coprinellus* correlated positively with UR, and *Fusarium* correlated positively with TDW and UNR. However, *Lecanicillium* and *Acrocalymma* correlated negatively with AN, AP, AK, MBC, MBN, TDW, RGR, NC, and UNR (Figure 7B). At the order level of bacteria, Gemmatimonadales, Betaproteobacteriales, Xanthomonadales, Cytophagales, and Myxococcales correlated positively with AN, TDW, RGR, NC, and UNR, and Microtrichales, Micromonosporales, Streptomycetales, and Micrococcales correlated negatively with AN, AP, AK, MBC, MBN, TDW, RGR, NC, and UNR (Figure 7C). At the genus level of bacteria, *Sphingomonas* correlated positively with AN, AP, AK, MBN, TDW, RGR, NC, and UNR. However, *Nocardioides*, *Novosphingobium*, and *Stackebrandtia* correlated negatively with AN, AP, AK, MBN, TDW, RGR, NC, and UNR (Figure 7D).

## Discussion

With deteriorating climate conditions, drought stress due to water scarcity is becoming increasingly severe, resulting in reduced crop yields and posing major challenges to global food security (Berdugo et al., 2020; Baker, 2021; Franco-Navarro et al., 2021). It is therefore urgent to explore new and effective ways of improving drought tolerance. Melatonin and dopamine play an important role in plant resistance to abiotic stress, so we explored their role in enhancing the drought tolerance of apples in greater depth.

### Regulation of growth and development by melatonin and dopamine

Water shortages caused by drought decrease the water potential in soil, which makes it difficult for plants to obtain the nutrients and water needed to maintain normal growth and development (Bhusal et al., 2021). In addition, the chlorophyll content declines with intensification of drought stress, which seriously reduces the photosynthetic efficiency of plants. These factors lead to a significant decrease in the accumulation of dry matter in plants, which seriously inhibits their growth and development. Melatonin and dopamine can significantly alleviate the degradation of chlorophyll, effectively promote the accumulation of dry matter, and thus significantly reduce the inhibitory effects of drought stress on plant growth and development (Supplementary Figure S1).

Drought stress causes ultrastructural changes in mitochondrial and chloroplast, resulting in excessive production of ROS, which can lead to lipid peroxidation and membrane dysfunction (Wang et al., 2017; Kanwar et al., 2018; Sharma et al., 2020). Mitochondria and chloroplasts are the organs that synthesize plant melatonin. These results suggest that mitochondria and chloroplasts will be the main battlefields for melatonin to remove ROS and alleviate oxidative stress in plants (Wang et al., 2017; Kanwar et al., 2018; Tan and Reiter, 2019). Dopamine can also effectively remove ROS and alleviate chlorophyll degradation, which suggests its similarity with melatonin. In addition, plants use their antioxidant systems to address this, with antioxidant enzymes playing significant roles (Balfagon et al., 2021). However, there is a time limit for antioxidant enzymes to respond to drought stress (Zhang et al., 2021). Under long-term drought stress, the activities of SOD, CAT, and APX in apple leaves initially increase and then decrease (Figure 1). As excellent antioxidants, melatonin and dopamine not only directly remove peroxides from plants but also activate the antioxidant enzyme system (Sharma et al., 2020). In this study, melatonin and dopamine enhanced the activities of SOD, POD, CAT, and APX to varying degrees and also significantly reduced the levels of reactive oxygen species (Figures 1; Supplementary Figure S2). They also significantly enhanced the expression of genes corresponding to these antioxidant enzymes under drought stress (Sharma et al., 2020). Moreover, they significantly increased the soluble sugar content, simultaneously increased the free proline content, regulated osmotic pressure, and improved plant drought tolerance (Supplementary Figure S3).

### Regulation of transport and metabolism of N by melatonin and dopamine

The absorption and utilization of mineral nutrients by plants is severely inhibited under drought stress, and the resulting nutrient deficit disrupts their normal physiological and biochemical activities (Liang et al., 2018b). Among these nutrients, N, which has the highest demand, is a key factor in maintaining normal growth and development (Du et al., 2022b). The results of isotope labeling showed that the accumulation of N in various parts of the plant and the uptake activity and utilization rate of N were significantly reduced under drought stress. In addition, the ratio of ^15^N in shoots to that in the whole plant decreased (Figure 2). These results suggest that drought stress severely restricts the uptake of ^15^N by roots and its transport from roots to shoots, thus significantly inhibiting growth and development. Both melatonin and dopamine can effectively promote the N absorption and utilization of plants under drought stress (Figure 2). In this study, the concentration, accumulation, and uptake activity of ^15^N in MDT roots were significantly higher than those in DDT, indicating that the ability of melatonin to promote N uptake in roots under drought stress was better than the ability of dopamine (Figure 2).

The forms of inorganic N absorbed by plants and transported to various organs are mainly NH_4_^+^ and NO_3_^−^, which are completed by *AMT* and *NRT* family transporters, respectively (Liu and von Wiren, 2017; Wang et al., 2018). Studies have confirmed that prolonged drought has a significant inhibitory effect on some *AMT* and *NRT* family genes (Liang et al., 2018b). Under drought stress, melatonin and dopamine significantly increased the expression levels of *NRT1*.*1*, *NRT2*.*4*, *NRT2*.*5*, *NRT2*.*7*, *AMT1*.*2*, *AMT1*.*5*, *AMT1*.*6*, and *AMT2*.*1* (Figure 3), which is consistent with the results of ^15^N isotope labeling, suggesting that melatonin and dopamine promote NH_4_^+^ and NO_3_^−^ uptake and transport in plants under drought stress. In addition, NH_4_^+^ and NO_3_^−^ not only provide the necessary requirements for growth and development of plants but also regulate activities as signal molecules (Liu and von Wiren, 2017; Wang et al., 2018). Recent studies have shown that applying the right amount of N can effectively mitigate the inhibition of long-term drought on plant growth and development (Cheng et al., 2021) and that ammonium is an important factor in drought resistance (Huang et al., 2018). More importantly, N is an important element in the synthesis of organic osmotic regulatory substances (proline, amide, protein, and so forth), which can significantly increase the abiotic stress tolerance of plants (Averina et al., 2014; Zhong et al., 2018). Melatonin and dopamine significantly upregulated the expression of genes associated with N metabolism and related enzyme activities (Figure 3C), increasing the proline content (Supplementary Figure S2B), demonstrating that these compounds also enhance drought tolerance by regulating N metabolism. Furthermore, among the 12 genes measured in this experiment, the three genes most affected by melatonin were *NR* (16.97-fold; at 30 days), *AMT2*.*1* (13.56-fold; at 30 days), and *NRT2*.*4* (5.36-fold; at 15 days), and the three genes most affected by dopamine were *AMT2*.*1* (11.96-fold; at 30 days), *NR* (8.13-fold; at 15 days), and *NRT2*.*4* (8.00-fold; at 15 days; Figure 3). These results suggest that there are similarities between melatonin and dopamine in regulating absorption, transport, and metabolism of N in plants under drought stress.

### Regulation of the environment of the rhizosphere microsphere by melatonin and dopamine and effect on the absorption and use of N

Over time, plant roots have evolved a complex assemblage of microbial communities that affect growth, nutrition, and health (Fitzpatrick et al., 2018). These symbiotic relationships between plants and microorganisms can significantly improve the adaptability of plants to various types of natural adversity and the nutrient absorption capacity (Jiang et al., 2017; Gao et al., 2020). Therefore, maintaining a good soil environment and microbial community structure is very important for plant growth and development and soil nutrient absorption and utilization, and it is also one of the important ways to enhance stress resistance (de Vries et al., 2020). Drought stress can lead to changes in microbial community structure, and the changes become gradually intensified over time (Santos-Medellin et al., 2021). In our study, melatonin and dopamine significantly increased the α diversity of fungi under drought stress, suggesting that the two compounds can play a positive role in the soil fungal community. Furthermore, the NMDS results showed that the degree of separation of the fungal community was higher than the degree of separation of the bacterial community in the different treatment (Figures 4, 5). These results suggest that melatonin and dopamine have a great effect on fungal community diversity.

At the phylum level of fungi, melatonin and dopamine significantly increased the relative abundances of Basidiomycota and Mortierellomycota in rhizosphere soils (Figure 6A). Basidiomycota mainly decompose lignin and are closely related to soil N cycling, and Mortierellomycota plays an important role in soil nutrient cycling (Zhang et al., 2011; Koch et al., 2021). Furthermore, we analyzed the abundance of flora at the order and genus levels. Dopamine significantly enhanced the relative average abundance of Saccharomycetales (Figure 6B), which has many beneficial effects on plant growth and the soil N, P, and S cycles (Ramya et al., 2021). The correlation analysis showed that Saccharomycetales was positively correlated with AN and NC. Under drought conditions, melatonin increased the relative average abundance of Mortierellales (Figure 6B), which was significantly positively correlated with UR in soil (Figure 7A). Moreover, both melatonin and dopamine can increase the abundance of *Mortierella* and *Penicillium* (Figure 6C), which were significantly positively correlated with UR (Figure 7B). Soil urease can catalyze the formation of NH_3_ from urea, which is related to the N cycle in soil, and its activity can reflect the N supply capacity of soil (Wang et al., 2020a). These results suggest that melatonin and dopamine may enhance soil urease activity by increasing the relative abundances of *Mortierella* and *Penicillium*, thus regulating soil N cycling. In addition, *Mortierella* plays a key role in soil phosphorus cycling (Miao et al., 2016) and *Penicillium* plays an important role in protecting the soil health of apple orchards (Faria et al., 2008), which contribute to nutrient cycling, maintaining a good rhizosphere environment under drought stress, and promoting plant growth and development.

There were also significant differences between the relative abundances of bacterial populations within the same treatments. Long-term drought stress reduced the abundance of Proteobacteria and increase the abundance of Actinobacteria (Figure 6D). These changes are similar to the effects of long-term drought stress on rhizosphere microorganisms of sorghum and rice (Santos-Medellin et al., 2021; Xu et al., 2021). Moreover, Actinobacteria play an important role in promoting plant growth, carbon metabolism, and nutrient transformation in soil and in iron absorption and utilization in plants (Faria et al., 2008; Barka et al., 2016). This suggests that plants cope with drought stress by regulating the microbial community structure of the rhizosphere. Primitive bacteria are thought to be the origin of the major melatonin-producing organs (mitochondria and chloroplasts) in plants. And the antioxidant function of melatonin is evolutionarily conserved in almost all organisms (Reiter et al., 2009; Tan et al., 2013; Tan and Reiter, 2020). However, only scattered studies have reported the melatonin synthesis potential of cyanobacteria and proteobacteria (Jiao et al., 2021). The abundance of Proteobacteria was elevated in MDT compared to DT, which may be explained by exogenous melatonin promoting the growth of bacteria capable of melatonin synthesis in the soil (Figure 6D). Furthermore, we explored the changes in bacterial communities at the order and genus levels. Gemmatimonadales can adapt to changes in the environment by regulating their carbon and N intakes (Li et al., 2017). However, drought stress can reduce the abundance of Gemmatimonadales, which was positively correlated with AN and NC (Figure 7C), and thus may inhibit the uptake of N by plants. Melatonin can improve the abundance of Betaproteobacteriales (Figure 6E), which can grow rapidly in a nutrient-rich environment, under drought stress (Trivedi et al., 2013). Correlation analysis showed that Betaproteobacteriales was significantly positively correlated with AN and MBC (Figure 7C). In addition, both melatonin and dopamine can significantly increase the relative abundances of Thermomicrobiales and IMCC26256 (Figure 6E). Both of these are significantly positively correlated with UR (Figure 7C). Melatonin also increased the relative abundance of *Sphingomonas* (Figure 6F), which was significantly positively correlated with AN, TDW, RGR, and NC (Figure 7D). Moreover, melatonin and dopamine significantly reduced the relative average abundances of *Stackebrandtia* and *Nocardioides* (Figure 6F), which were significantly negatively correlated with AN, TDW, RGR, and NC (Figure 7D).

Taken together, our results show that melatonin and dopamine improve the content of available N in soil by improving the microbial community structure of fungi and bacteria and thus promoting the absorption and utilization of N in plants and maintaining the growth and development of plants under drought stress. Moreover, they significantly reduce the value of 16S/ITS, which makes the bacterial and fungal diversity of the rhizosphere microbial community more balanced (Supplementary Figure S5). Studies have shown that the fungi/bacteria ratio can, to some extent, reflect the response of the food network structure and function in soil to different soil conditions and that the ecosystem is more stable when the fungi/bacteria ratio is higher (de Vries et al., 2006).

## Conclusion

**Figure 8:**
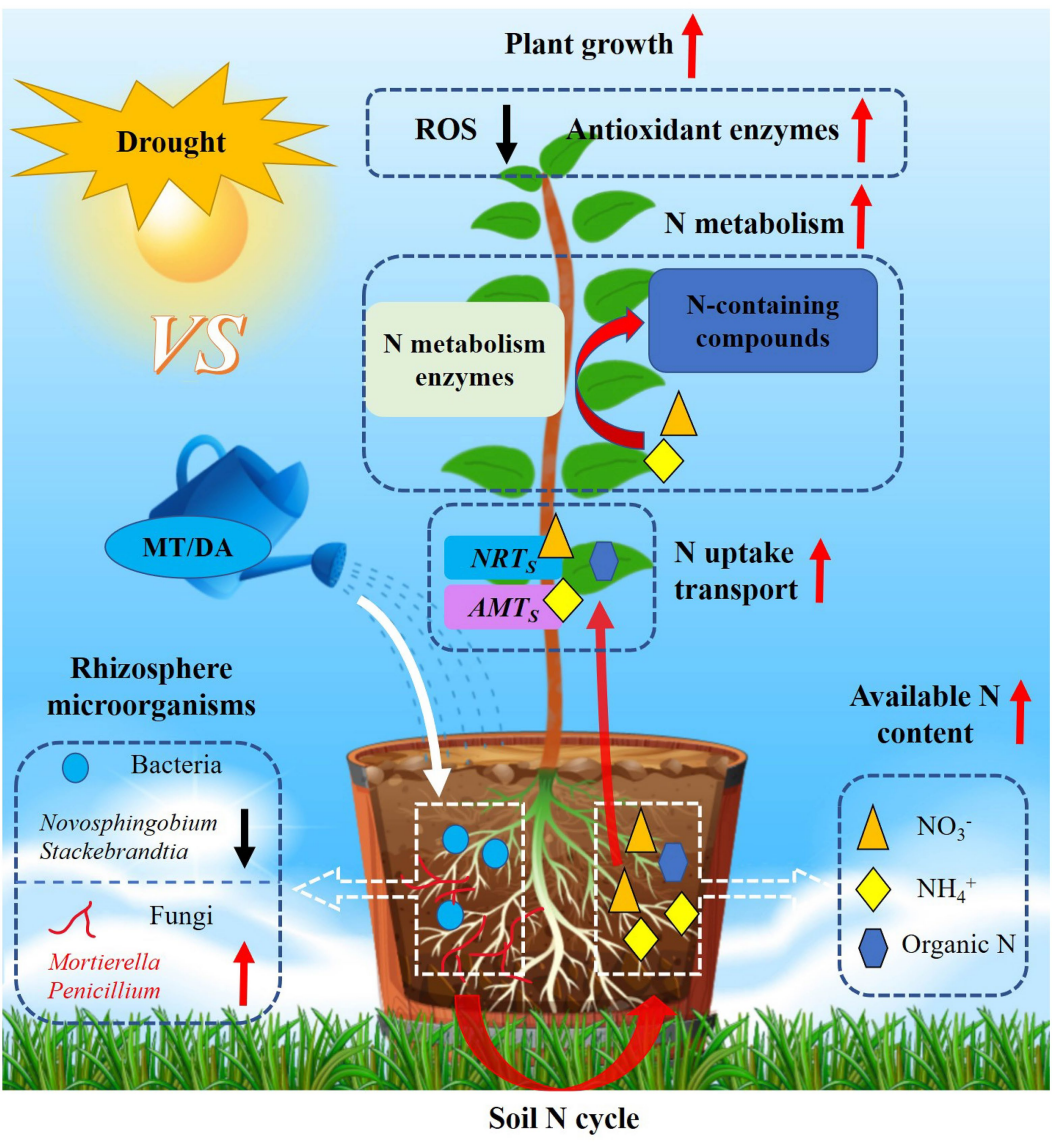
Proposed model for the potential mechanisms of melatonin and dopamine mitigation of drought stress. Under drought conditions, melatonin and dopamine applied in soil can enhance plant drought tolerance by removing reactive oxygen species, promoting N absorption and utilization, and changing the composition of rhizosphere microbial community. Melatonin and dopamine can exert positive (red arrow) and negative (black arrow) effect on a variety of plant and soil indices.

This study confirmed that melatonin and dopamine can maintain continuous and efficient operation of antioxidant enzyme systems; regulate the expression of genes related to N uptake, transportation, and metabolism; and shape the rhizosphere microbial community structure of apple under drought stress. Related analyses showed that these compounds affect the content of available N in soil and also urease activity by adjusting the richness of some rhizosphere microbial populations (negative: *Novosphingobium*, *Stackebrandtia*; positive: *Mortierella*, *Penicillium*), thereby improving the uptake and utilization efficiency of N by plants and enhancing drought resistance (Figure 8). By elucidating the important roles of melatonin and dopamine in the apple plant–soil environment–soil microbial community composite association network, this study supports the future development of drought-resistant and water-saving cultivation of apple.

## Data availability statement

The datasets presented in this study can be found in online repositories. The names of the repository/repositories and accession number(s) can be found at: NCBI SRA - SRP388303.

## Author contributions

BL, PD, and YC conceived and designed the experiments. PD and YC performed the experiments with assistance from BY, SZ, ZL, and XZ. PD and YC analyzed the data and wrote the paper. BL provided financial support and helped perform the analysis with constructive discussions. JX provided materials and laboratory apparatus. All authors contributed to the article and approved the submitted version.

## Funding

This work was supported by the National Natural Science Foundation of China (Project No. 31901964), the Natural Science Foundation of Hebei (Project No. C2021204158), the Science and Technology Project of Hebei Education Department (Project No. BJK2022012), and the Introduced Talents Project of Hebei Agricultural University (Project No. YJ201904).

## Conflict of interest

The authors declare that the research was conducted in the absence of any commercial or financial relationships that could be construed as a potential conflict of interest.

## Publisher’s note

All claims expressed in this article are solely those of the authors and do not necessarily represent those of their affiliated organizations, or those of the publisher, the editors and the reviewers. Any product that may be evaluated in this article, or claim that may be made by its manufacturer, is not guaranteed or endorsed by the publisher.

## Footnotes

^1^ http://www.omicsmart.com
