# Supplementary material for: Improved tolerance of apple plants to drought stress and nitrogen utilization by modulating the rhizosphere microbiome via melatonin and dopamine
Source: Front Microbiol. 2022 Nov 10;13:980327. doi: 10.3389/fmicb.2022.980327 (PMC9687389; doi:10.3389/fmicb.2022.980327)
Supplement: Supplementary file 1 [file Data_Sheet_1.docx]

**Supplemental Table 1** Sequences of primers used in quantitative real-time RT-PCR

| Gene | Primer squence (5'-3') |
| --- | --- |
| *AMT1:2* F | AAGCGAGGATGAGACTCAAGGG |
| *AMT1:2* R | GCAGGACTGGCATCATTAACAGG |
| *AMT1:5* F | ACAGATAGTGGTAATTATAGGGTGGGT |
| *AMT1:5* R | CGTGGTCATGGTACACGTAAGC |
| *AMT1:6* F | GTAACTTATTGTTCACCTCTGGGGC |
| *AMT1:6* R | CGTTCCTGAACGCGTCGAAT |
| *AMT2:1* F | GTGACGATGGATCGATTGAGACTC |
| *AMT2:1* R | CCCGCTAACAAAATAAGAGTAATAGCT |
| *NRT1:1* F | CTGGCTGGTCCCACAGTTCTT |
| *NRT1:1* R | CTTCATTCCTTTCGGGCACTC |
| *NRT2:4* F | CAGAAGGTGAACCCGGAAG |
| *NRT2:4* R | CAAGTGGAACGTCCTCATGTG |
| *NRT2:5* F | TTGTGGTCCATCTAAGAACAAGGC |
| *NRT2:5* R | TCATCAGAGGGTCGGGTAACAG |
| *NRT2:7* F | TCTCCAGGCAGACGAGCATT |
| *NRT2:7* R | GGAGCAAGTGATACTGGTTTGTTTC |
| *NR* F | CGATGACGACGAGAATGAGGAC |
| *NR* R | GCGGACCATAGACGAGTTACGAC |
| *GS* F | ATATCTGCTGGAGATGAACTGTGG |
| *GS* R | TGGACTTGGTGCTGTAGTTTGTG |
| *Fd-GOGAT* F | CGAAGGAAGAAGAAGACCACGC |
| *Fd-GOGAT* R | TTGCTGGTGCCTGTTGGGTT |
| *β-Actin* F | GGATTTGCTGGTGATGATGCT |
| *β-Actin* R | AGTTGCTCACTATGCCGTGCT |


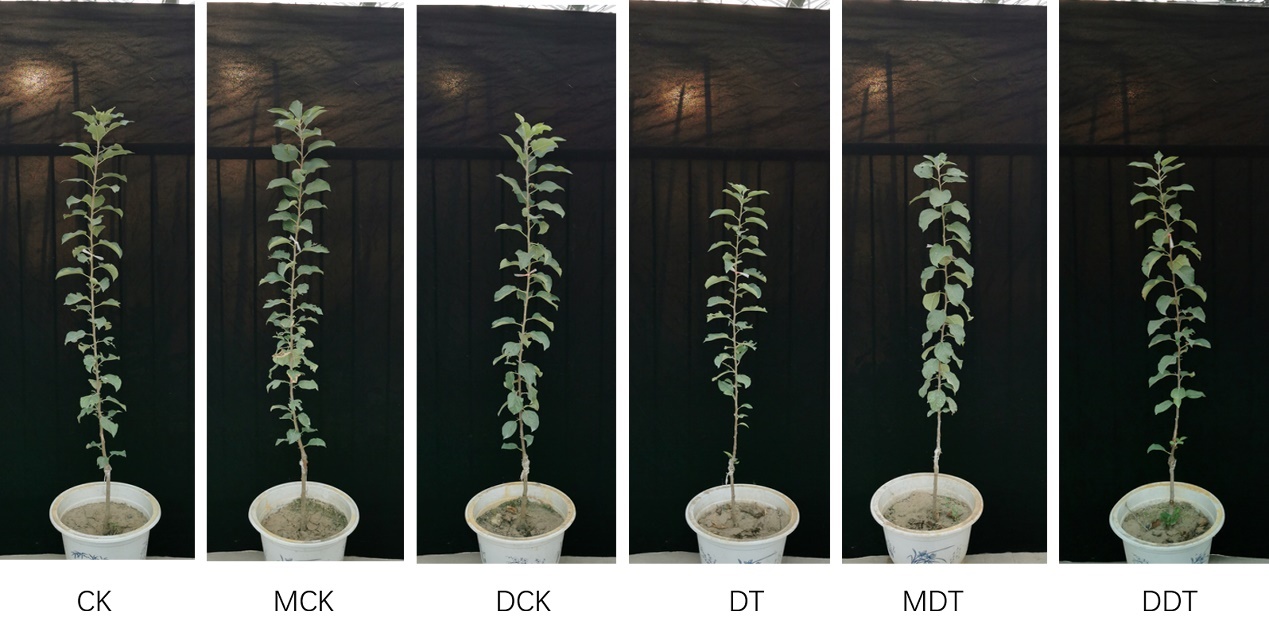


**Supplemental Figure 1|** Plant phenotype after 60 days of drought treatment.


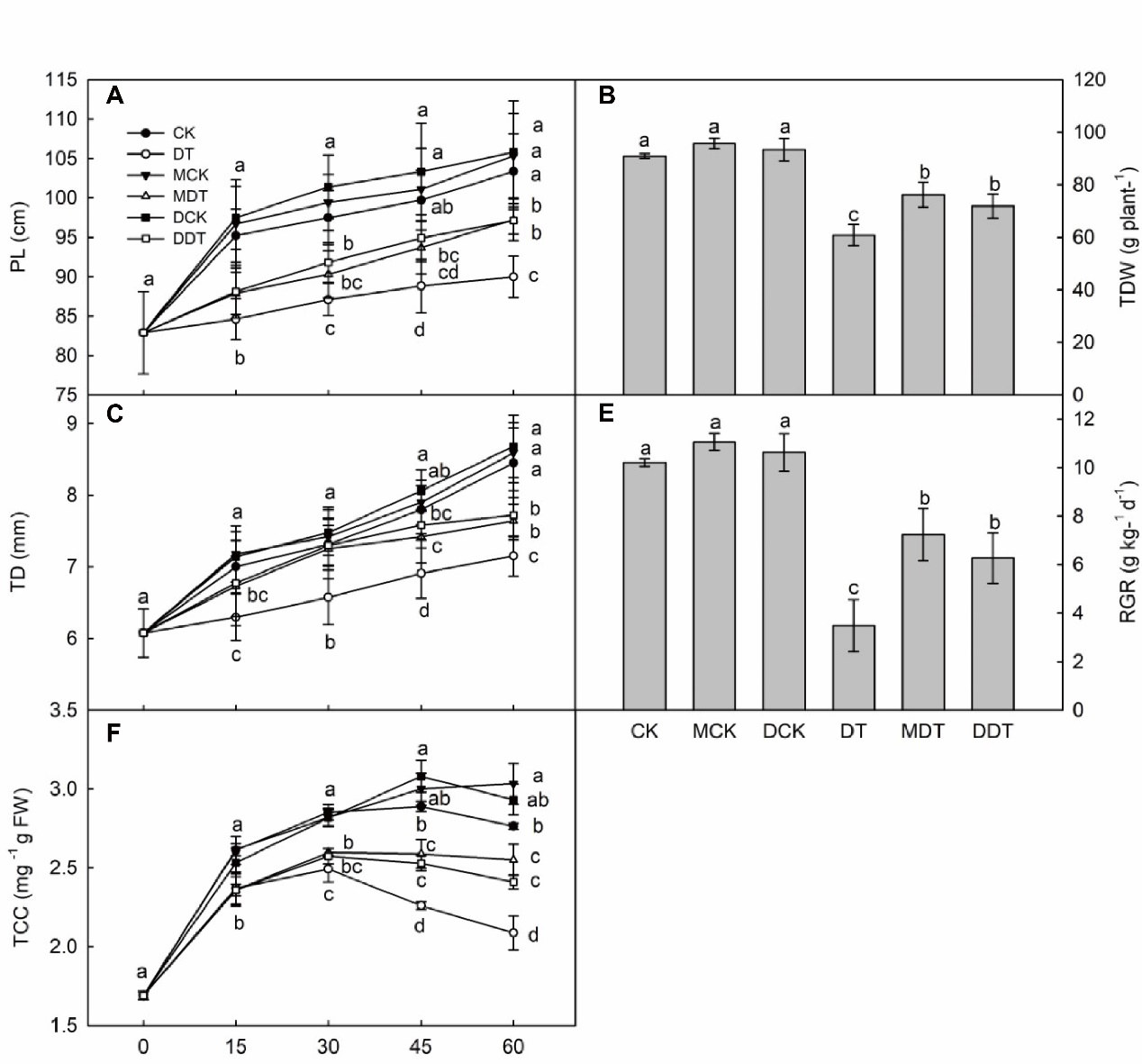


**Supplemental Figure 2|** Effect of melatonin or dopamine on plant length (PL), trunk diameter (TD) and total chlorophyll concentration (TCC) during 60d drought treatment, and total dry weight (TDW), relative growth rate (RGR) after 60d of drought treatment. Data are means ± SD of 10 replicate samples. The significance of differences between Tukey’s multiple-range tests with different letters representing the graph base (*P*<0.05).


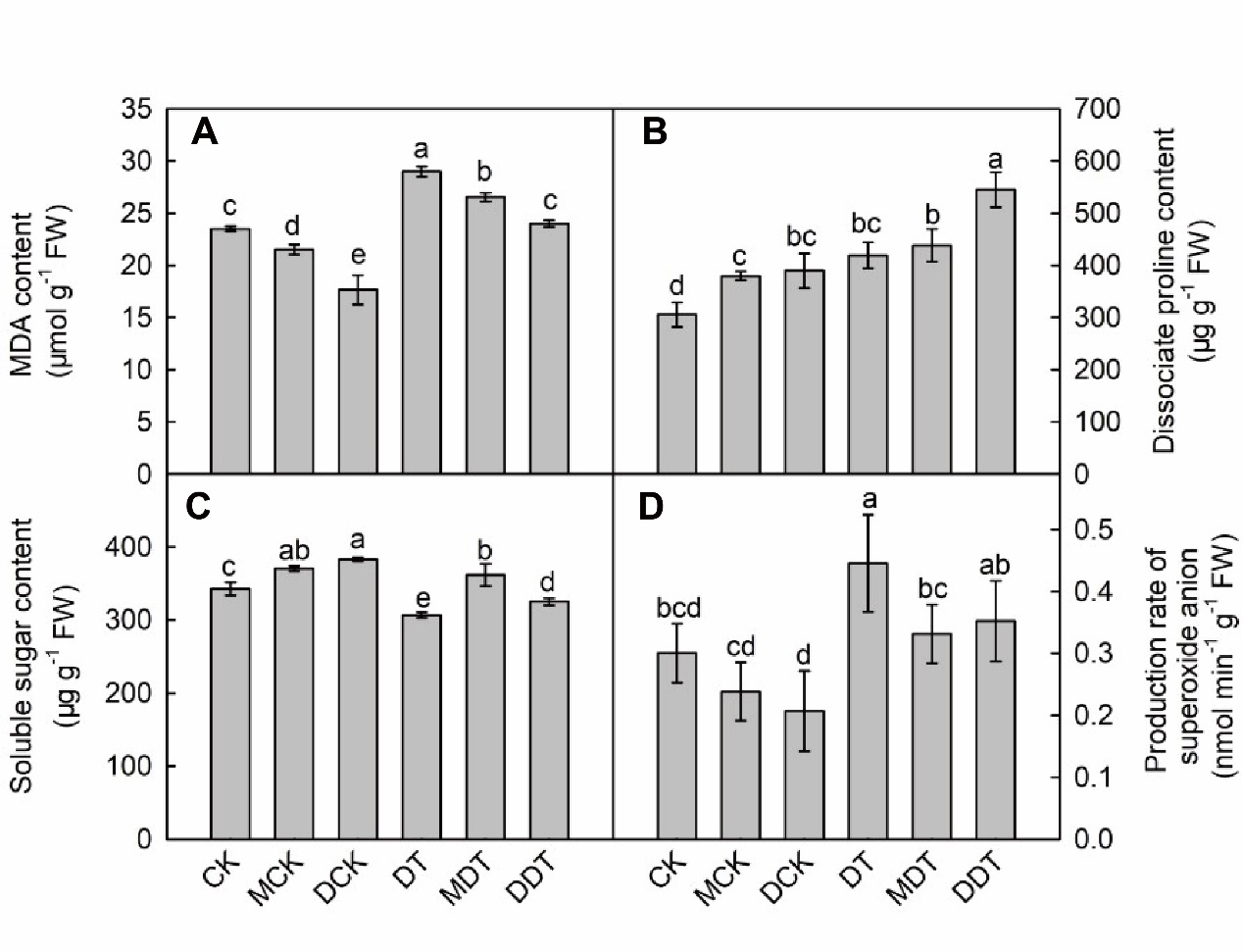


**Supplemental Figure 3|** Effect of melatonin or dopamine on Malondialdehyde (MDA), total soluble sugars, proline content and production rate of superoxide anion after 60d of drought treatment. Data are means ± SD of 5 replicate samples. The significance of differences between Tukey’s multiple-range tests with different letters representing the graph base (*P*<0.05).


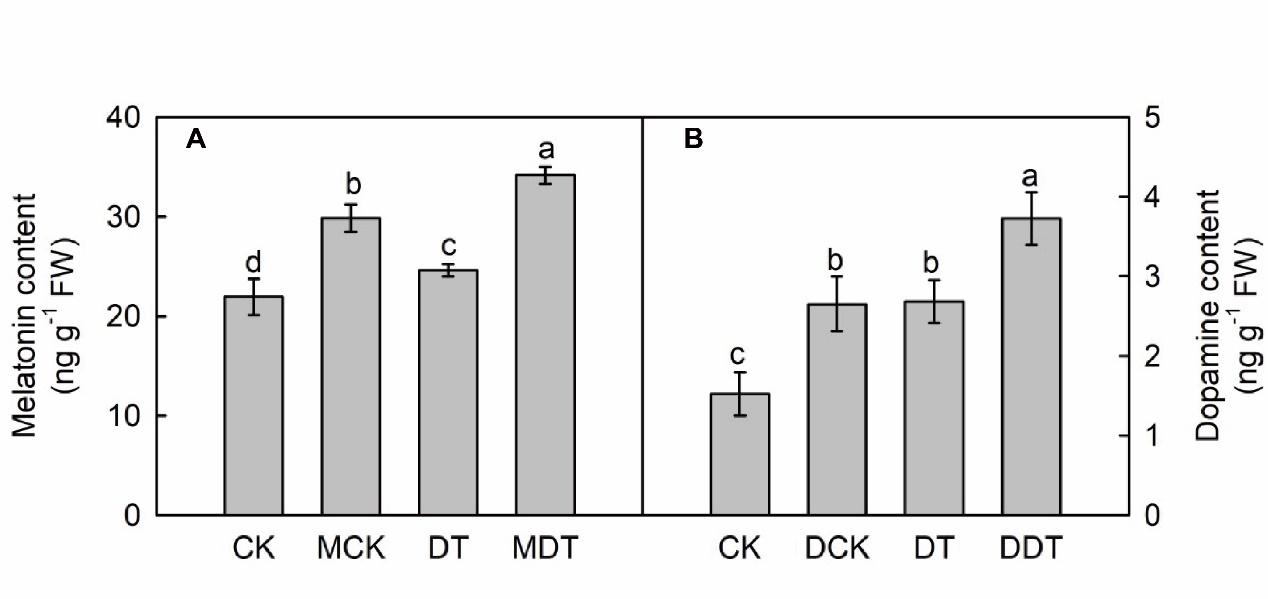


**Supplemental Figure 4|** Endogenous levels of melatonin and dopamine in plant leaves after 60 days of different treatments. Data are means ± SD (n = 3). For each panel, bars not labeled with same letter indicate significant differences at *P*<0.05, based on Tukey’s multiple range tests.


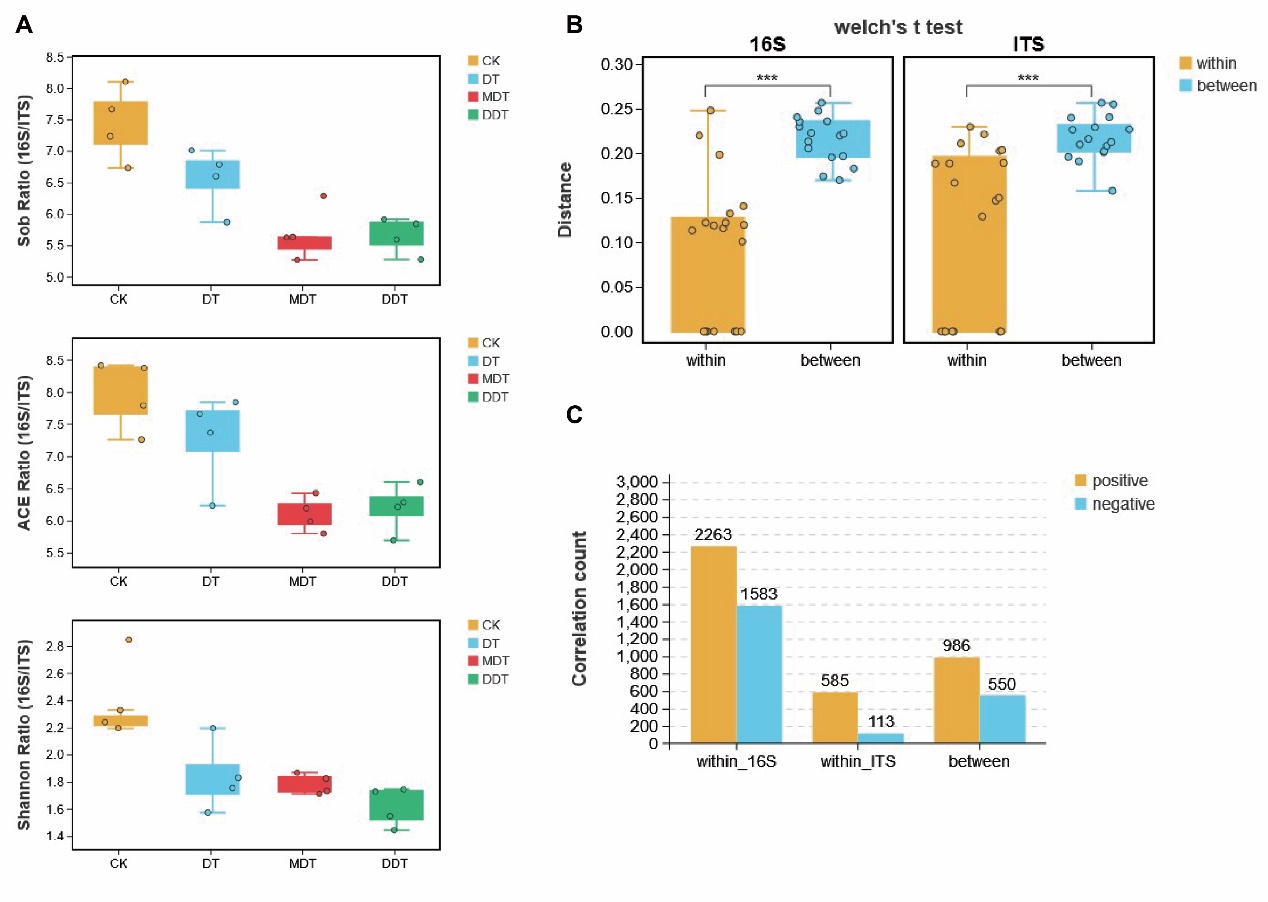


**Supplemental Figure 5|** Combined analysis of fungi and bacteria.
